# Genetic markers of lipid metabolism genes associated with low susceptibility to HCV infection

**DOI:** 10.1038/s41598-019-45389-4

**Published:** 2019-06-21

**Authors:** Luis Miguel Real, Juan Macías, Antonio Rivero-Juárez, Francisco Téllez, Dolores Merino, Sonia Moreno-Grau, Adelina Orellana, Juan Gómez-Salgado, María E. Sáez, Mario Frías, Anaïs Corma-Gómez, Nicolás Merchante, Agustín Ruiz, Antonio Caruz, Juan A. Pineda, GEHEP 012 study group, Marta Fernández-Fuertes, María Iglesias, Pilar Rincón

**Affiliations:** 1 Unidad Clínica de Enfermedades Infecciosas y Microbiología, Hospital Universitario de Valme, Sevilla, Spain; 2 Departamento de Bioquímica, Biología Molecular e Inmunología, Facultad de Medicina, Universidad de Malaga, Málaga, Spain; 3 Instituto Maimónides de Investigación Biomédica de Córdoba (IMIBC), Hospital Universitario Reina Sofía de Córdoba, Universidad de Córdoba, Cordoba, Spain; 4 Hospital Universitario de Puerto Real, Instituto de Investigación e Innovación de la Provincia de Cádiz, Cádiz, Spain; 5 Unidad de Enfermedades Infecciosas, Hospital Universitario Juan Ramón Jiménez, Huelva, Spain; 6 Fundació ACE-Institut Català de Neurociències Aplicades, Universidad Internacional de Catalunya (UIC), Barcelona, Spain; 7 Universidad Espiritu Santo, Guayaquil, Ecuador; 8 Centro Andaluz de Estudios Bioinformáticos (CAEBI, SL), Sevilla, Spain; 9 Unidad de Inmunogenética, Universidad de Jaén, Jaén, Spain

**Keywords:** Clinical genetics, Prognostic markers

## Abstract

Due to the relation between lipids and Hepatitis C virus (HCV) life-cycle, we aimed to explore the existence of single nucleotide polymorphisms (SNPs) associated with low susceptibility to HCV-infection within lipid metabolism genes. This was a case-control study in three phases: (I) allelic frequencies of 9 SNPs within 6 genes were compared in 404 HCV-infected patients and 801 population controls; (II) results were validated in 602 HCV-infected individuals and 1352 controls; (III) results were confirmed in 30 HCV-exposed uninfected (EU) individuals. In phase I, only the *LDLRAP1*-rs4075184-A allele was differentially distributed in patients and controls (358 of 808 alleles [44.3%] and 807 of 1602 alleles [50.3%], respectively) (p = 0.004). In phase II, the A allele frequency was 547 of 1204 alleles (45.4%) in patients and 1326 of 2704 alleles (49.0%) in controls (p = 0.037). This frequency in EU was 36 of 60 alleles (60%), which was higher than that observed in patients from phase I (p = 0.018) and phase II (p = 0.027). The *LDLRAP1*-mRNA expression was lower in AA carriers than in non-AA carriers (median [Q1-Q3]: 0.85 [0.17–1.75] relative-units [ru] versus 1.71 [1.00–2.73] ru; p = 0.041). Our results suggest that *LDLRAP1*-rs4075184-A allele is associated with lower susceptibility to HCV-infection and with reduced expression of *LDLRAP1*-mRNA.

## Introduction

Few individuals exposed to the hepatitis C virus (HCV) remain uninfected without seroconversion^1^.

The low susceptibility or total resistance to HCV-infection could be determined genetically. As a biological proof of principle, an *in vitro* study reported that three naturally occurring single nucleotide polymorphisms (SNPs) in the peptidyl-prolyl isomerase A (*PPIA)* gene abrogated HCV replication^2^. In spite of this, no subject homozygous for the minor alleles of these mutations was found in a cohort of HCV-exposed uninfected (EU) individuals^3^.

Because of the intimate relation between the lipids and viral life cycle^4^, variations in genes encoding for proteins related to lipid metabolism could be involved in the susceptibility to HCV infection. In fact, it has been recently observed that the lipidomics profiling of EU individuals is distinct from HCV-infected individuals, even those who clear viremia, either spontaneously or after antiviral therapy^5^. However, there are scarce genetic association studies focused in genes involved in the lipid metabolism in this setting^6^. Specifically, rs5925^7^ and rs688^8^ genetic markers within *LDLR* gene as well as rs1042034^9^ polymorphism linked to *APOB* gene have been associated with HCV-infection susceptibility. But none of them has been validated so far. The identification of genetic markers related to the HCV-infection susceptibility in genes involved in the lipid metabolism could help to understand some aspects of the virus life cycle as well as, to design new preventive or therapeutic strategies against HCV-infection.

Because of this, the aim of this study was to explore the existence of genetic factors associated with the low susceptibility to HCV infection within genes related to the lipid metabolism.

## Results

### Study populations

A total of 3251 individuals were included in this work to perform genetic association analyses in the three phases.

In phase I, the HCV-infected group I was originally composed of 442 HCV-chronically infected individuals. Among them, 38 (8.6%) were discarded because they showed genetic relationships with other individuals included in this group. Therefore, this dataset was finally constituted by 404 individuals. The control group I was constituted by 801 individuals.

In phase II, only 609 (48.4%) subjects from a total of 1258 HCV-chronically infected individuals attended in our Unit since 1999 had an available sample. Among them, 7 (1.1%) individuals were discarded because their ethnicity could not be probed. Therefore, 602 individuals were finally included in the HCV-infected group II. The control group II was constituted by 1352 individuals randomly selected from 6000 individuals collected in the genome research program at ACE foundation. The 1352 controls were those necessary to have 80% power to detect differences in allelic frequencies taking into account (i) 15% of genotype losses, (ii) the number of individuals included in the HCV-infected group II and, (iii) the difference in allelic frequencies described in phase I for the rs4075184 in controls and HCV-infected individuals.

In phase III, a total of 30 individuals were recruited as EU. All of these EU individuals were HIV-infected and users of illegal drugs during a median (Q1-Q3) of 11.5 (9.0–13.5) years. During that period, they reported to share injection devices during 6 (4.0–8.25) months. Moreover, 62 (52.5%) individuals from 118 subjects who were unequivocally identified as cases of spontaneous clearance of HCV infection were also included in this study.

Main characteristics of all those individuals are depicted in Table 1.

**Table 1:** Main characteristics of the study populations.

Variables	HCV-infected group I (n = 404)	Control group I (n = 801)	HCV-Infected group II (n = 602)	Control group II (n = 1352)	Spontaneous Resolvers (n = 62)	Exposed uninfected (n = 30)
Age, years^†^	41.8 (38.3–45.4)	50.9 (45.9–58.0)	49.5 (45.7–53.2)	64 (57.0–70.0)	43.0 (40.0–45.0)^‡^	49.5 (43.5–54.0)
Male gender	323 (80.0)	433 (54.1)	500 (83.0)	412 (30.5)	49 (79.0)	24 (80.0)
HIV coinfection, n (%)	302 (74.8)		375 (62.3)		39 (62.9)	100 (100)
**Risk factors for infection**						
Parenteral drug users, n (%)	334 (82.7)		485 (88.5)		46 (74.2)	100 (100)
MSM^§^, n (%)	27 (6.7)		14 (2.3)		6 (9.7)	
Blood transfusion, n (%)	9 (2.2)		9 (1.5)		1 (1.6)	
Not known, n (%)	34 (8.4)		94 (15.6)		9 (14.5)	
**HCV genotype**						
1a, n (%)	50 (12.3)		200 (33.2)			
1b, n (%)	51 (12.6)		116 (19.3)			
1 a/b, n (%)	8 (2.0)		5 (0.8)			
1 (unknown subtype), n (%)	107 (26.5)		19 (3.2)			
2, n (%)	2 (0.5)		5 (0.8)			
3, n (%)	130 (32.2)		134 (22.3)			
4, n (%)	56 (13.9)		98 (16.3)			
Not known, n (%)	0 (0)		25 (4.1)			

^†^Median (quartil 1- quartil 3).

^‡^Data available in 51 individuals.

^§^Men who have sex with men.

### Phase I. Discovery

A total of 81 SNPs linked to genes involved in the lipid metabolism were selected from those previously genotyped in HCV-infected group I. Among them, only 16 (19.7%) SNPs linked to *LDLRAP1*, *PCSK9*, *HMGCR*, *LPL*, *VLDLR* and *LDLR* genes had genotype data in the control group I (Supplementary Table 3). However, the genotypic distribution of those 7 SNPs linked to *LDLR* gene, which were in high LD (d′ > 0.9) (Supplementary Fig. 2), were not in accordance with the HWE law in the control group I (Supplementary Table 4) and, consequently, they were discarded. Finally 9 (11.1%) SNPs were available for genetic association studies. The genotypic distributions and allelic frequencies of these SNPs in the control group I and in the HCV-infected group I are depicted in Table 2.

**Table 2:** Genotypic distribution and allelic frequencies of genetic markers in the phase I.

CHR	SNP	Gene	A1	A2	Genotipic distribution in HCV-infected group I (A1A1/A1A2/A2A2)	Genotipic distribution in control group I (A1A1/A1A2/A2A2)	A1 frequency in HCV-infected group I (%)	A1 frequency in control group I (%)	P^†^
1	rs6687605	*LDLRAP1*	A	G	81/203/117	202/403/196	45.51	50.37	0.024
**1**	**rs4075184**	***LDLRAP1***	**A**	**G**	**77/204/123**	**202/403/196**	**44.30**	**50.37**	**0.004**
1	rs11563	*LDLRAP1*	A	C	77/205/121	202/403/196	44.54	50.37	0.006
1	rs7552841	*PCSK9*	A	G	78/197/127	143/389/265	43.90	42.34	0.466
1	rs603247	*PCSK9*	G	A	9/111/282	19/231/547	16.04	16.87	0.605
5	rs12654264	*HMGCR*	A	T	60/183/161	90/371/306	37.50	35.91	0.449
8	rs328	*LPL*	G	C	6/79/319	13/176/609	11.26	12.65	0.323
9	rs7032549	*VDLDLR*	G	A	91/210/103	157/413/231	48.51	45.38	0.145
9	rs10812379	*VDLDLR*	G	A	38/179/184	81/348/361	31.79	32.27	0.811

CHR, Chromosome; A1, Allele 1; A2, Allele 2; *LDLRAP1*, Low density lipoprotein receptor adaptor protein 1; *PCSK9*, Protein convertase subtilisin/kexin-type 9; *HMGCR*, 3-Hydroxi-3-methylgluraryl-CoA reductase; *LPL*, Lipoprotein lipase; *VDLDLR*, very low density lipoprotein receptor.

^**†**^Calculated by PLINK software using standard case/control allelic association analysis.

Only the SNP rs4075184 showed a significant different allelic distribution between the two groups after multiple testing correction. Specifically, there was a lower frequency of the A allele in the HCV-infected population (358 out of 808 alleles) than in the control population (807 out of 1602 alleles) (p = 0.004, OR = 0.78, 95%CI = 0.66–0.93) (Table 2).

**Figure 1:**
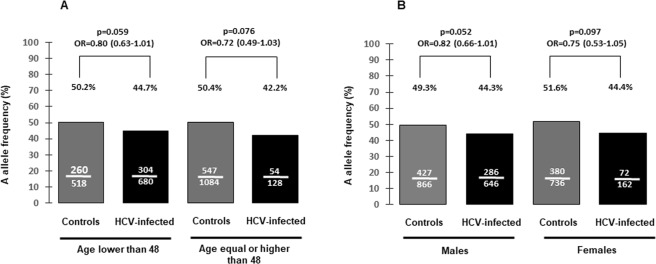
Comparison of rs4075184 A allele frequencies in controls and in HCV-infected individuals from phase I stratified by age 48 (**A**) or by gender (**B**).

To test if these differences were affected by age or gender, we performed separated stratified studies by gender or by the median age of the entire population included in phase I (48 years). Differences in the allelic frequencies on each strata did not reach the statistical significance. Moreover, the ORs and the allelic frequencies did not vary more than 8% from that observed in the study performed in the entire population (Fig. 1). After data adjustment by age the OR (95%CI) was 0.78 (0.64–0.94) (adjusted p = 0.012). The corresponding value after data adjustment by gender was 0.79 (0.67–0.95) (adjusted p = 0.013).

### Phase II. Validation

To validate the result obtained in phase I, the rs4075184 genetic marker was genotyped in a different sample of population controls (control group II) and HCV-infected patients (HCV-infected group II). The genotypic distributions in controls and HCV-infected individuals were 340 (25.1%) AA, 646 (47.8%) AG and 366 (27.1%) GG; and, 132 (22.0%) AA, 283 (47.0%) AG, and 187 (31%) GG, respectively (HWE p value > 0.10 in both groups). Accordingly, the frequency of rs4075184 A allele was 49.3% (1326 out of 2704 alleles) in the control group II and 45.4% (547 out of 1204 alleles) in the HCV-infected group II (p = 0.037, OR = 0.86, 95%CI = 0.76–0.99).

### Phase III. Confirmation

**Figure 2:**
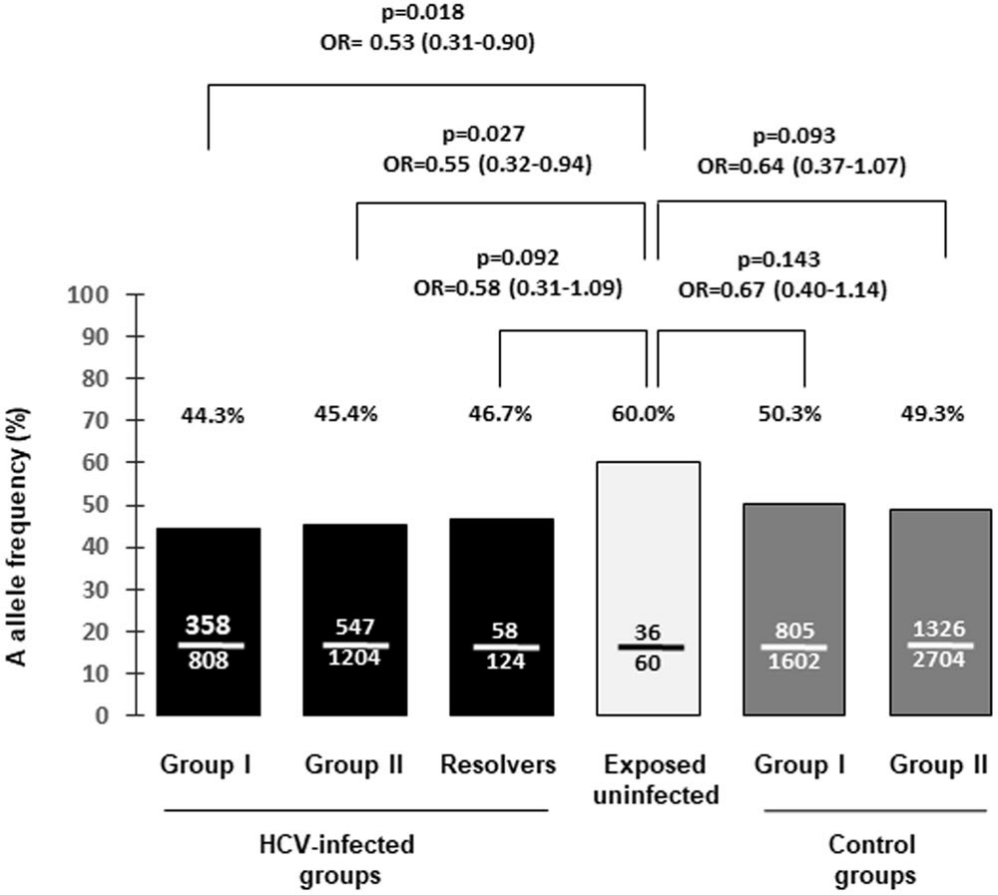
Comparison of rs4075184 A allele frequencies in controls groups, HCV-infected groups, HCV spontaneous resolvers and exposed uninfected individuals.

To confirm the association of rs4075184 with the low susceptibility to HCV infection we analysed the genotypic distribution of this marker in 30 EU individuals. Nine (30%) individuals were AA, 18 (60.0%) were AG, and 3 (10%) were GG (HWE p value > 0.100). The frequency of rs4075184 A allele in this sample was 60.0% (36 out of 60 alleles). This frequency was significantly higher than that observed in the HCV-infected group I or that reached in the HCV-infected group II (Fig. 2). The frequencies of the rs4075184 A allele in EU were also numerically higher than those observed in both control groups, but the differences were not statistically significant (Fig. 2).

To explore if the rs4075184 A allele could be related to spontaneous viral clearance, this marker was also genotyped in 62 spontaneous resolvers of HCV infection. Among them, 14 individuals (22.6%) were AA, 30 (48.4%) were AG, and 18 (29.0%) were GG (HWE p value > 0.800). The A allele frequency was similar to those found in both HCV-infected groups. Although this frequency was not statistically different to that observed in EU individuals there was a trend to a significant statistical association (Fig. 2).

### *LDLRAP1* exons sequencing analyses

The coding region of *LDLRAP1* gene was bi-directionally sequenced in 17 EU individuals: 9 homozygous for the rs4075184 A allele, 7 heterozygous and 1 individual with the GG genotype. There were two common SNPs; rs6687605 and rs28969504 located in exon 6 and 7 respectively, that showed complete LD with the rs4075184 genetic marker (d′ = 1). It was not detected any variation related to dysfunction of the LDLRAP1 protein.

### *LDLRAP1* mRNA expression analysis

**Figure 3:**
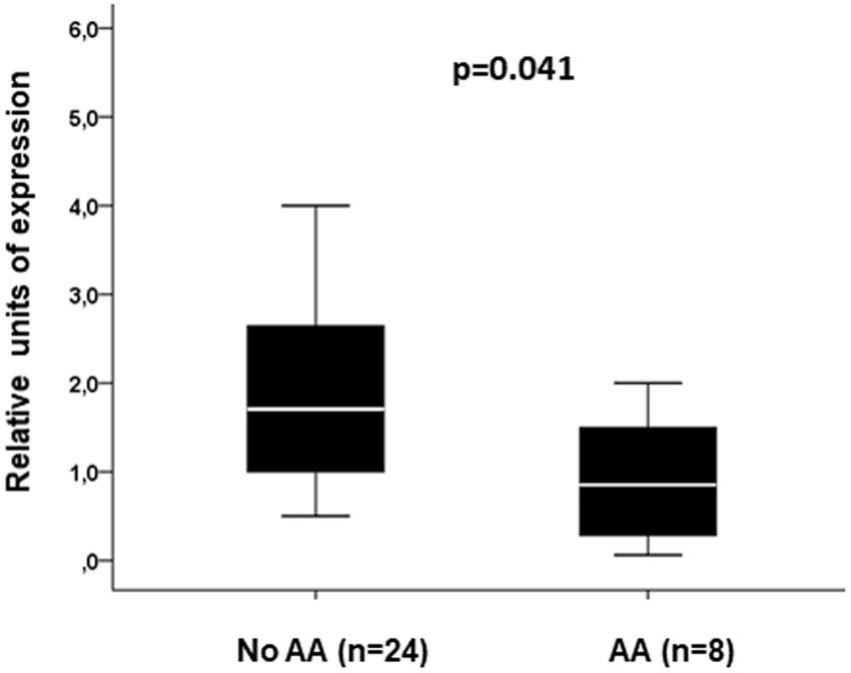
*LDLRAP1* mRNA expression in peripheral blood mononuclear cells from healthy people according to the genotype of rs4075184.

The expression of *LDLRAP1* mRNA gene in PBMCs was analysed in 32 anonymous voluntaries. Among them, the genotypic distribution of rs4075184, was 8 (25%) AA, 16 (50%) AG and 8 (25%) GG (HWE p value = 1.000). All these individuals were non-related healthy Spanish subjects. The expression of *LDLRAP1* mRNA was lower in individuals carrying the AA genotype than in those carrying the AG or GG genotype (median [Q1-Q3]: 0.85 [0.17–1.75] relative units [ru] versus 1.71 [1.00–2.73] ru; respectively) (p = 0.041) (Fig. 3).

## Discussion

In this study, we found a genetic association of *LDLRAP1* rs4075184 A allele with the low susceptibility to HCV infection and with reduced expression of *LDLRAP1* mRNA.

LDLRAP1 is an adaptor that interacts with the cytoplasmic tail of low density lipoprotein receptor (LDLR), phospholipids, and component of the clatrin endocytic machinery^10^. LDLRAP1 is recruited to the plasma membrane after LDL binding facilitating endocytosis of the LDL-LDLR complex in hepatocytes^11^. Consequently, mutations in this gene can cause hypercholesterolemia (OMIM*605747).

LDLR acts as HCV-LDL complex receptor. Although, the role of LDLR in post-binding HCV entry is controversial^12,13^, its involvement in HCV infection is well established^13,14^. In addition, LDLR expression is stimulated by the HCV to promote the lipid uptake and facilitate viral proliferation. Consequently, it has been suggested that patients with familial hypercholesterolemia due to mutations in the *LDLR* gene may be protected against HCV infection^15^. Indeed, polymorphisms linked to *LDLR* have been associated with Hepatitis C viral load^16^ and with the capacity for responding to interferon-based antiviral therapy^17–20^. Thus, it could be hypothesized that a dysfunction of LDLRAP1 would affect to the capacity of LDLR to favour the entry of HCV or, alternatively, it could interfere with the metabolic requirements of the HCV life cycle.

We found a lower expression of *LDLRAP* mRNA in those individuals carrying the rs4075184 AA genotype. This finding was in accordance with that reported in the Genotype-Tissue Expression (GTEX) project (dbGAP Accesion phs000424.v7.p2) (www.gtexportal.org/home/snp/rs4075184). This fact could be the functional basis of the observed genetic association in this study. Interestingly, this SNP seems not related to spontaneous HCV clearance. It does not necessarily deny the role of *LDLRAP1* expression in the low susceptibility to HCV infection, but it suggests the possible existence of other unknown mechanisms involved in this setting.

A possible explanation for the association observed is that rs4075184 A allele could be in LD with other causative SNP that would not be present in HCV-infected individuals but present in EU individuals. To explore this hypothesis we sequenced the coding region of the *LDLRAP1* gene, mainly in those rs4075184 AA EU individuals. Nevertheless, we did not find any genetic alteration that could be related to LDLRAP1 dysfunction. In spite of this, we cannot discard the existence of mutations linked to rs4075184 A allele in other DNA regions that could confer protection against the HCV-infection. More functional experiments and DNA sequencing analyses are necessary to explain the basis of the association observed.

Polymorphisms within genes related to the lipid metabolism have been previously associated with low susceptibility to HCV infection such as the rs5925 and rs688 within *LDLR* gene^7,8^ and the rs1042034 within *APOB* gene^9^. Unfortunately, none of them were included in our study and, consequently, we were not able to validate those findings. Since these SNPs, as well as rs4075184, belong to genes involved in LDL and HCV uptake, it would be very interesting to explore whether there is a genetic interaction between them for better explaining the low susceptibility to HCV infection.

Recently, SNPs linked to *RELA*^21^ and *CD209*^22^ genes have been related to the low HCV-infection susceptibility. Although these works, as the ours, need replication in other series, taken together they suggest that resistance to HCV may be a multi-genetic trait, as was also suggested for resistance to HIV-infection^23^.

This study has some limitations. First, there were differences in the distribution of age and gender among groups of cases and controls included in phase I and II. Case/control allelic association analysis is more powerful than other ones, but it does not allow adjusting by covariables. To overcome this limitation we performed stratified analyses by age and gender in phase I. In these studies, the odds ratios obtained were almost identical to those obtained in the entire population. Indeed, in spite of the differences in age and gender between all groups, the rs4075184 A allele frequency was very similar among the two groups of controls, as well as among in the two groups of HCV-infected individuals included in both I and II phases, suggesting that neither age nor gender are confounding variables. Second, due to the scarce number of EU individuals, the sample size of these subjects in our study, like in others^22^, was relatively small to carry out genetic association analyses. However, they constituted a high quality population as HCV EU taking into account the high prevalence of coinfection among parenteral drug users infected by HIV in our population^24^. This is a strength of our work. Besides, in phase I and II we employed an indirect strategy to identify genetic markers associated with low susceptibility to HCV infection. Thus, we assumed that if a genetic factor has a role in the protection against viral infection, it should be differentially represented in the infected population with respect to its frequency in the general population. This strategy has been previously used by others in the setting of HIV or HCV infection susceptibility^7,9,23^. The inclusion of a sample of EU individuals as a confirmation phase enforces our findings. This is another strength of our study. Third, the inclusion of HCV-infected individuals in phase II was conditioned to the existence of stored blood or DNA samples. Similarly, individuals included as controls in phase II were aged individuals to be used as controls in genetic analyses focussed in Alzheimer’s disease. Therefore, selection bias cannot be excluded. In spite of this, (i) the homogeneity of the allelic frequencies obtained in this phase with respect to the same groups included in phase I and, (ii) the fact that *LDLRAP1* has not been previously associated with Alzheimer´s disease (according to the GWAS catalogue data from NHGRI-EBI available at www.ebi.ac.uk/gwas), suggest that the allelic frequencies obtained in phase II are not deviated. Finally, the analysis of the *LDLRAP1* expression was carried out in peripheral blood mononuclear cells (PBMCs). Because the target cells of HCV are hepatocytes, these analyses should have been performed in liver tissue. In spite of this, they were performed in a relative large number of people obtaining robust results.

In conclusion, our results suggest a role of *LDLRAP1* gene in the low susceptibility to HCV-infection. Because the protective allele is associated with a reduced mRNA expression of *LDLRAP1* gene, and taking into account that we did not found any DNA mutation in the coding sequence of this gene in EU individuals, this could be the mechanism underlying this protection against HCV infection. However, further studies are needed to explore the exact role of *LDLRAP1* in this setting. If our results are confirmed, LDLRAP1 could be considered a target for preventing the HCV infection in individuals with high-risk habits.

## Material and Methods

### Design

This was a retrospective case-control study developed in three phases: discovery (phase I), validation (phase II) and confirmation (phase III). All individuals included in this study were non-related Spanish Caucasians.

### Phase I

#### Patients and controls

Two genetic data sets from two groups of individuals were used in phase I: control group I and the HCV-infected group I. The control group I was constituted by a sample of the Spanish population which was recruited by a random sampling approach from a cross-sectional population-based epidemiological survey performed in eight different cities of Spain as previously described^25^. More details about the recruitment of these subjects are described elsewhere^26^. The HCV-infection status of these individuals was not known. All of them had been genotyped using the Affymetrix Nsp I 250 K chip (Affymetrix, Santa Clara, USA)^25^. In this group a high number of genotypes were also imputed as described elsewhere^27^. These individuals have been used as controls in several genome-wide association studies (GWASs)^27–29^. The HCV-infected group I was constituted by HCV chronically infected individuals who consecutively initiated a pegylated interferon plus ribavirin treatment from May 2000 to December 2010 in the Infectious Diseases Units of 5 tertiary care hospitals in Spain and who were genotyped for 116 SNPs^16,17,30^.

#### Single nucleotide polymorphisms selection

From the two available datasets, all those SNPs linked to genes related to the lipid metabolism that were previously genotyped in the HCV-infected group I and that were also genotyped or imputed in the control group I were selected (see supplementary Table 3). Genotypic data from these SNPs were extracted from those datasets using Plink software^31^. Those SNPs whose genotypic distribution did not fulfil the Hardy-Weinberg equilibrium law (p < 0.05) in any of these groups were discarded.

#### Statistical analyses

The program Graphical Representation of Relationships (GRR)^32^ was used to check sample relatedness and to correct potential sample mislabelling, duplications, or contaminations in phase I. Thus, due to the differences in the amount of SNPs genotyped in the two datasets, those individuals who showed an identity by state (IBS) > 0.1875 with other individuals in the control group I, or an IBS > 1.750 with other individuals in the HCV-infected group I, were removed.

Plink software^31^ was used to carry out standard case/control allelic association (1 degree of freedom) as well as to perform the Hardy-Weinberg equilibrium (HWE) test in phase I. In this phase, multiple testing correction was applied taking into account the number SNPs selected. Thus, the p-value threshold was established by the following formula: p = 0.05/number of SNPs. Finally, 9 SNPs were selected, establishing the p-value cut-off for statistical significance at 0.005. For the rest of comparisons the p-value threshold was set at 0.05.

Haploview software (version 4.1) (https://www.broad.harvard.edu/haploview/haploview) was employed to define the linkage disequilibrium (LD) blocks using the ‘solid spine of LD’ algorithm. Pair-wise LD was measured with Lewontin’s standardized deviation coefficient (d′).

The stratified analyses in phase I to calculate adjusted p and odds ratios (OR) values were performed by mean of Mantel-Haenszel test using the statistical calculator Statcalc from Epi Info™ 7 (available at https://www.cdc.gov/epiinfo/index.html).

### Phase II

#### Patients and controls

Two groups of individuals were included in phase II: Control group II and HCV-infected group II. Control group II was constituted by individuals randomly selected from those that are being collected since 2012 under the genome research program of the ACE foundation (http://www.fundacioace.com/grace/) to be used as controls in genome wide association studies (GWASs) focussed in Alzheimer’s disease. The HCV-infection of these individuals was not known. All of them were cognitively healthy according to the neuropsychological test battery (NBACE) used in Fundació ACE^33^. More details about the recruitment of these subjects are described elsewhere^34^.

The HCV-infected group II was constituted by those individuals not included in the HCV-infected group I who were attended since 1999 due to a chronic HCV infection in the Infectious Disease Units of our Hospital and who received or not therapy against Hepatitis C. They were included if they had available a frozen blood or a DNA sample.

#### DNA isolation and genotyping

DNA was isolated from frozen whole blood samples using the Quick Pure Blood DNA extraction Kit (Macherey-Nagel, Düren, Germany), Magna Pure system (Roche Diagnostics, Mannheim, Germany) or Qiacube system (Qiagen, Hilden, Germany) in accordance with the manufacturers’ instructions.

The rs4075184 genetic marker was genotyped using the LightCycler 480 instrument (Roche Diagnostics, Mannheim, Germany). Amplification primers and sequence-specific probes are depicted in Supplementary Table 1. Real-time PCRs conditions and melting curve analyses were performed as previously described by Martinez-Mir *et al*.^35^ except for annealing temperature that was at 61 °C.

#### Statistical analyses

The sample size of the control group II was calculated with the ENE 3.0 software (GlaxoSmithKline, Madrid, Spain).

For the comparison of rs4075184 allelic frequencies between groups and to carry out the HWE test, we used the online resource at the Institute for Human Genetics, Munich, Germany (http://ihg.gsf.de).

### Phase III

#### Exposed uninfected individuals and spontaneous resolvers of HCV infection

Those HIV-infected individuals who reported to have been using parenteral drugs with shared injection devices for longer than three months were included as EU subjects if they were seronegative for HCV. EU individuals are being collected from four tertiary hospital in Spain since 2016. Besides, individuals identified as spontaneous resolvers of HCV infection since 1999 in our hospital and who had available a frozen blood or a DNA sample were also included as an additional control sample. Spontaneous resolvers were considered to those individuals with positive anti-HCV antibodies who did not show HCV-ARN in plasma and who were never treated against HCV.

#### DNA isolation, genotyping and DNA sequencing

DNA was isolated from frozen whole blood samples using the Qiacube system (Qiagen, Hilden, Germany) in accordance with the manufacturers’ instructions.

The rs4075184 genetic marker was genotyped as described above.

Purified PCRs products covering the 9 exons of *LDLRAP1* and part of the 5′ and 3′ untranslated regions (Supplementary Fig. 1) were bi-directionally sequenced using standard capillary electrophoresis techniques. Primers used for DNA amplification, which were the same used for sequencing, are depicted in Supplementary Table 2. All PCRs were performed in 20 μL of reaction volume containing 20 ng of DNA, 0.5 μM of each primer, 1x My Taq reaction buffer (Bioline, London, UK) and 0.5 Units of My Taq DNA Polymerase (Bioline). PCR conditions were as follows: denaturation at 95 °C for 3 min, followed by 35 cycles at 95 °C for 60 s, 61 °C for 60 s, and 72 °C for 60 s, and a final extension at 72 °C for 3 min. Purification of PCR products were performed using the Illustra ExoProstar (GE Healthcare, Chicago, Illinois, USA) in accordance with manufacturer’s instructions.

#### Statistical analyses

Data were analysed as described in phase II.

### LDLRAP1 mRNA quantification

A total of 32 anonymous individuals from the staff of our hospital, who voluntarily donate a blood sample for participating in this study, were used to perform mRNA expression analyses. All of them were genotyped for the rs4075184 genetic marker as described above.

Total RNA was purified from 5 × 10^6^ of PBMCs using Tripure reagent (Roche). RNA was quantified by spectrophotometry and dissolved at 1 μg/μl. Reverse transcription of total RNA was performed using the Superscript III (Invitrogen), following manufacturer’s instructions. All the RNA samples were also subjected to control reactions without reverse transcriptase to check for potential contamination. *LDLRAP1* mRNA expression was normalized by the human β-actin mRNA expression as an endogenous control. Both quantitative PCRs were carried out in the same tube using the *LDLRAP1* Taqman gene expression Assay ID Hs00296701_m1 (Applied Biosystems, Waltham, Massachusetts, USA) and the Human ACTB (Beta Actin) endogenous control (Applied Biosystems), following the manufacturer’s instructions. Each cDNA sample was amplified in triplicate wells. Parallel negative control experiments were done with reverse transcriptase negative cDNAs. The relative expression of *LDLRAP1* mRNAs compared to endogenous β-actin controls were calculated using the MX-pro V4.0 software (Agilent Technologies, Santa Clara, California, USA) according to manufacturer’s instructions.

### Ethics

This study was in compliance with the national legislation and it was performed according to the ethical guidelines of the Declaration of Helsinki. The study was approved by the Ethics Committee of the Hospital Universitario de Valme (internal reference number: 0422-N-16). Informed consent was obtained from all individuals before sampling.

## Supplementary information

Supplementary information

## Data Availability

All data generated or analysed during this study are included in this published article (and its Supplementary Information files).
