# Supplementary material for: Genetic markers of lipid metabolism genes associated with low susceptibility to HCV infection
Source: Sci Rep. 2019 Jun 21;9:9054. doi: 10.1038/s41598-019-45389-4 (PMC6588564; doi:10.1038/s41598-019-45389-4)

Genetic markers of lipid metabolism genes associated with low susceptibility to HCV infection

Luis Miguel Real[1,2;*], Juan Macías[1], Antonio Rivero-Juárez[3], Francisco Téllez[4], Dolores Merino[5], Sonia Moreno-Grau[6], Adelina Orellana[6], Juan Gómez-Salgado[7], María E Sáez[8], Mario Frías[3], Anaïs Corma-Gómez[1], Nicolás Merchante[1], Agustín Ruiz[6], Antonio Caruz[9], Juan A Pineda[1]. On behalf of GEHEP 012 study group

[1] Unidad Clínica de Enfermedades Infecciosas y Microbiología. Hospital Universitario de Valme, Sevilla, Spain. [2] Departamento de Bioquímica, Biología Molecular e Inmunología. Facultad de Medicina. Universidad de Malaga. Málaga, Spain. [3] Instituto Maimónides de Investigación Biomédica de Córdoba (IMIBC). Hospital Universitario Reina Sofía de Córdoba. Universidad de Córdoba. Cordoba, Spain. [4] Hospital Universitario de Puerto Real . Instituto de Investigación e Innovación de la Provincia de Cádiz, Cádiz, Spain. [5] Unidad de Enfermedades Infecciosas. Hospital Universitario Juan Ramón Jiménez. Huelva, Spain. [6] Fundació ACE-Institut Català de Neurociències Aplicades. Universidad Internacional de Catalunya (UIC). Barcelona, Spain. [7] Universidad de Guayaquil. Guayaquil, Ecuador. [8] Centro Andaluz de Estudios Bioinformáticos (CAEBI, SL). Sevilla, Spain. [9] Unidad de Inmunogenética, Universidad de Jaén. Jaén, Spain.

Supplementary table 1. Amplification primers and probes for rs4075184 genotyping.

**Polymorphism**	**rs4075184**
Forward primer	TAAGGGTGATGACCTCTGCC
Reverse primer	AACAGCAGAGAACAGGCTCG
Anchor probe	Cy5-CACCCGTCATCCAGATTCCC-Ph
Sensor probe	AAAATTAGTACAGAGAATTCCTGTT-Flc

Cy5, Fluorochrome Cy5; Flc, Fluorescein; Ph, Phosphorothionate. All sequences given in 5´ to 3´ direction.

Supplementary table 2. Primers used for the amplification of *LDLRAP1* exons.

Exon covered	Forward primer†	Reverse Primer†	Amplicon size (bases)
1	AAACTCTTGGTGCCTGGACC	CTAGCTGGCCAGGACAGC	669
2	AAGAAGGCTGGTGAGAGCTG	CCAAAAGCTGGATGGAAGG	364
3	TTGCAAGAAGGTGCTGGCTG	GATGAGGAAACTGAGGCTCC	397
4	GGGAATAGCAGGTTCTCTGC	CTACTGCCCACACAAAGCTG	443
5 and 6	AGACCTGGAGAAGAACAGCC	CAAGTGAAAAACCAAGAGGCTC	939
7	CAGTCTAAGCCATTAGTCAGG	AGAGGCCAAGCATTGTGAGG	568
8	AGTCCCTGTAGCTTACCC	ATGGGACTGTTGCTCTGG	262
9	GGAGGTGCTTTGATCTGAGG	TCAATGCAGCACCATGGAGG	971

†All sequences given in 5´ to 3´ direction.

Supplementary table 3. Genetic markers selected from the HCV-infected group I and those that had been imputed/genotyped in the control group I.

Chr	SNP	gene	Available SNPs In Control group I	Chr	SNP	gene	Available SNPs In Control group I
1	rs6687605	*LDLRAP1*	rs6687605	9	rs1869592	*VLDLR*	
1	rs28969504	*LDLRAP1*		9	rs10812379	*VLDLR*	rs10812379
1	rs4075184	*LDLRAP1*	rs4075184	9	rs4740698	*VLDLR*	
1	rs11563	*LDLRAP1*	rs11563	15	rs1800588	*LIPC*	
1	rs17111503	*PCSK9*		16	rs1800775	*CETP*	
1	rs2479408	*PCSK9*		19	rs1529729	*LDLR*	
1	rs2479409	*PCSK9*		19	rs10415811	*LDLR*	
1	rs10888896	*PCSK9*		19	rs8102273	*LDLR*	
1	rs4927193	*PCSK9*		19	rs9305020	*LDLR*	
1	rs499718	*PCSK9*		19	rs11672123	*LDLR*	
1	rs10888897	*PCSK9*		19	rs6511720	*LDLR*	
1	rs676297	*PCSK9*		19	rs2228671	*LDLR*	
1	rs572512	*PCSK9*		19	rs12983082	*LDLR*	rs12983082
1	rs2495477	*PCSK9*		19	rs11669576	*LDLR*	
1	rs2479413	*PCSK9*		19	rs1003723	*LDLR*	rs1003723
1	rs7552841	*PCSK9*	rs7552841	19	rs5930	*LDLR*	
1	rs557435	*PCSK9*		19	rs4508523	*LDLR*	
1	rs483462	*PCSK9*		19	rs2738446	*LDLR*	rs2738446
1	rs603247	*PCSK9*	rs603247	19	rs688	*LDLR*	rs688
1	rs10465832	*PCSK9*		19	rs2738452	*LDLR*	rs2738452
1	rs662145	*PCSK9*		19	rs5925	*LDLR*	rs5925
2	rs3749054	*APO-B*		19	rs2738456	*LDLR*	
2	rs673548	*APO-B*		19	rs2738457	*LDLR*	
2	rs2854725	*APO-B*		19	rs2569540	*LDLR*	
2	rs11126598	*APO-B*		19	rs2738459	*LDLR*	
2	rs10199768	*APO-B*		19	rs2738460	*LDLR*	
2	rs11676704	*APO-B*		19	rs2116898	*LDLR*	
2	rs579826	*APO-B*		19	rs6413504	*LDLR*	rs6413504
2	rs531819	*APO-B*		19	rs14158	*LDLR*	
2	rs512535	*APO-B*		19	rs2738464	*LDLR*	
2	rs7575840	*APO-B*		19	rs2738465	*LDLR*	
5	rs12654264	*HMGCR*	rs12654264	19	rs1433099	*LDLR*	
8	rs328_LPL	*LPL*	rs328	19	rs2738466	*LDLR*	
9	rs1454626	*VLDLR*		19	rs7258950	*LDLR*	
9	rs7043199	*VLDLR*		19	rs4804570	*LDLR*	
9	rs7852409	*VLDLR*		19	rs4804146	*LDLR*	
9	rs7032549	*VLDLR*	rs7032549	19	rs405509	*APOE*	
9	rs7022122	*VLDLR*		19	rs429358	*APOE*	
9	rs10967213	*VLDLR*		19	rs439401	*APOE*	
9	rs1545566	*VLDLR*		19	rs445925	*APOE*	
9	rs4741747	*VLDLR*		19	rs4420638	*APOE*	

Chr, Chromosome; SNP, Single Nucleotide Polymorphism; *LDLRAP1*, Low density lipoprotein receptor adaptor protein 1; *PCSK9*, Protein convertase subtilisin/kexin-type 9; *APOB*, Apolipoprotein B; *HMGCR*, 3-Hydroxi-3-methylgluraryl-CoA reductase; *LPL*, Lipoprotein lipase; *VDLDLR*, very low density lipoprotein receptor; *LIPC*, Hepatic lipase; *CETP*, Cholesteryl ester transfer protein plasma; *LDLR*, Low density lipoprotein receptor, *APOE*, Apolipoprotein E.

Supplementary table 4. Genotypic distribution of genetic markers in cases and controls from phase I and Hardy-Weinberg equilibrium test.

Chr	Gene	SNP	A1	A2	Genotypic distributions in cases A1A1/A1A2/A2A2	HWE p value	Genotypic distributions in controls A1A1/A1A2/A2A2	HWE p value
1	LDLRAP1	rs6687605	A	G	81/203/117	0.762	202/403/196	0.887
1	LDLRAP1	rs4075184	A	G	77/204/123	0.687	202/403/196	0.887
1	LDLRAP1	rs11563	A	C	77/205/121	0.614	202/403/196	0.887
1	PCSK9	rs7552841	A	G	78/197/127	0.919	143/389/265	1.000
1	PCSK9	rs603247	G	A	9/111/282	0.713	19/231/547	0.447
5	HMGCR	rs12654264	A	T	60/183/161	0.524	90/371/306	0.182
8	LPL	rs328	G	C	6/79/319	0.618	13/176/609	0.873
9	VLDLR	rs7032549	G	A	91/210/103	0.485	157/413/231	0.285
9	VLDLR	rs10812379	G	A	38/179/184	0.645	81/348/361	0.870
19	LDLR	rs12983082	C	A	92/202/109	1.000	192/360/226	0.044
19	LDLR	rs1003723	A	G	92/203/107	0.842	192/360/226	0.044
19	LDLR	rs2738446	G	C	85/200/119	1.000	192/360/226	0.044
19	LDLR	rs688	A	G	86/198/120	0.841	192/360/226	0.044
19	LDLR	rs2738452	G	A	86/199/117	0.920	192/360/226	0.044
19	LDLR	rs5925	A	G	119/200/85	1.000	192/360/226	0.044
19	LDLR	rs6413504	G	A	96/208/99	0.551	192/360/226	0.044

CHR, Chromosome; A1, Allele 1; A2, Allele 2; HWE, Hardy-Weinberg equilibrium; *LDLRAP1*, Low density lipoprotein receptor adaptor protein 1; *PCSK9*, Protein convertase subtilisin/kexin-type 9; *HMGCR*, 3-Hydroxi-3-methylgluraryl-CoA reductase; *LPL*, Lipoprotein lipase; *VDLDLR*, very low density lipoprotein receptor; *LDLR*, Low density lipoprotein receptor.

Supplementary figure 1. Genetic structure of *LDLRAP1*, sequenced regions and position of the rs4075184 within the gene.

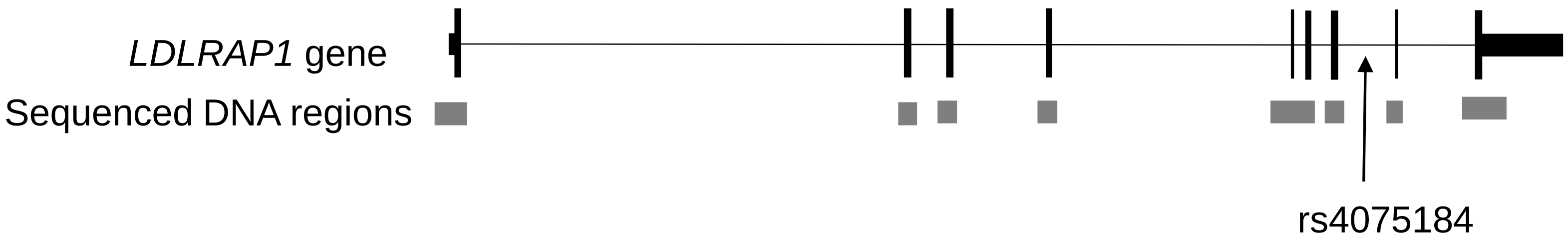

Supplementary figure 2. Linkage disequilibrium between those genetic markers that were genotyped in HCV-infected group I and in control group I. Numbers in shaded diamonds are d’ values that were obtained taken into account the entire population included in the phase I.

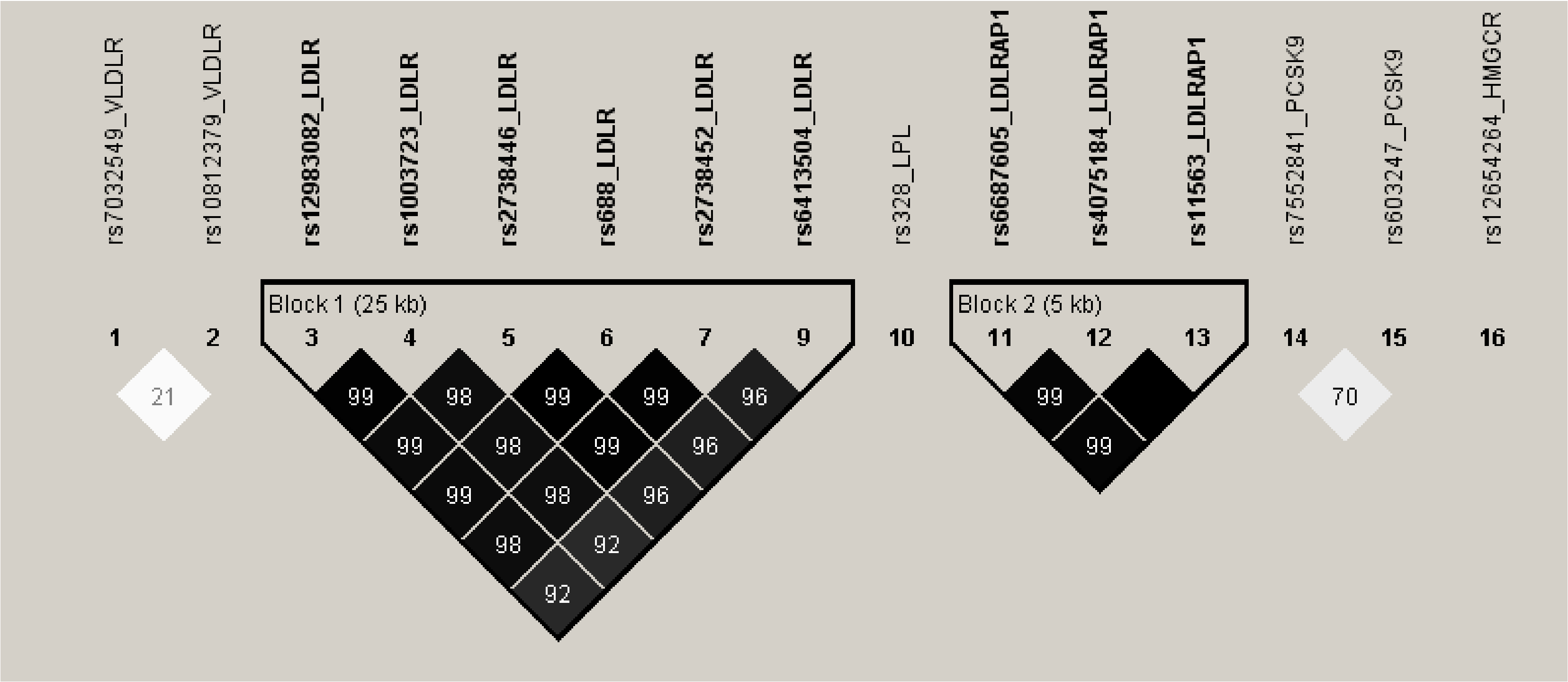

Supplement: Supplementary file 1 — Supplementary information [file 41598_2019_45389_MOESM1_ESM.pdf]
